# Infection of a Lepidopteran Cell Line with Deformed Wing Virus

**DOI:** 10.3390/v12070739

**Published:** 2020-07-09

**Authors:** Tal Erez, Nor Chejanovsky

**Affiliations:** Department of Entomology Institute of Plant Protection, Agricultural Research Organization, Rishon Lezion 7528809, Israel; taler@volcani.agri.gov.il

**Keywords:** Deformed wing virus, honey bee virus, Lepidopteran cells

## Abstract

Many attempts to develop a reliable cell cultured-based system to study honey bee virus infections have encountered substantial difficulties. We investigated the ability of a cell line from a heterologous insect to sustain infection by a honey bee virus. For this purpose, we infected the Lepidopteran hemocytic cell line (P1) with Deformed wing virus (DWV). The genomic copies of DWV increased upon infection, as monitored by quantitative RT-PCR. Moreover, a tagged-primer-based RT-PCR analysis showed the presence of DWV negative-sense RNA in the cells, indicating virus replication. However, the DWV from infected cells was mildly infectious to P1 cells. Similar results were obtained when the virus was injected into *Apis mellifera* pupae. Thus, though the virus yields from the infected cells appeared to be very low, we show for the first time that DWV can replicate in a heterologous cell line. Given the availability of many other insect cell lines, our study paves the way for future exploration in this direction. In the absence of adequate *A. mellifera* cell lines, exploring the ability of alternative cell lines to enable honey bee virus infections could provide the means to study and understand the viral infectious cycle at the cellular level and facilitate obtaining purified isolates of these viruses.

## 1. Introduction

Many viruses are associated with the honey bee *A. mellifera* (reviewed in [1]). Among them, Acute bee paralysis virus (ABPV), Deformed wing virus (DWV), Israel acute paralysis virus (IAPV), Kashmir bee virus (KBV), Sacbrood virus (SBV), Slow bee paralysis virus (SBPV), Chronic bee paralysis virus (CBPV), Apis mellifera filamentous virus (AmFV), and Black queen cell virus (BQCV) can induce severe pathogenicity to honey bees [2,3,4,5,6,7,8,9]. ABPV, CBPV, DWV, IAPV, and SBV can inflict severe damage to honey bee colonies, leading to their loss [3,4,5,8]. Understanding the course of the virus infection is crucial to develop the means to alleviate and\or suppress its damage to individual honey bees and colonies. To achieve this goal, experimental approaches were developed, including the extensive monitoring of virus infections in honey bee colonies [10,11,12,13,14,15], virus infectivity assays, the artificial infections of honey bees in cages [7,16,17], experimental colonies [8,18], the development of infectious viral clones [19,20], and fluorescent-labelled tagged viruses [21,22].

Cell lines are a crucial tool in virology to study in depth the cycle of virus replication and to obtain purified virus isolates [23,24]. Their use in animal virology rendered important insights in understanding the pathways of virus infections, the virus’ appropriation of the cellular machinery to its own advantage, and the interaction between the host-cell defenses and virus counter-means to avoid them [25,26]. This knowledge was efficiently channeled to develop anti-viral treatments [27].

Various attempts were made to infect honey bee cultured cells with some of the above-mentioned honey bee viruses. The *A. mellifera* cell line AmE-711 [28] was infected with a mixture of the honey bee viruses BQCV, DWV, IAPV, and SBV, [29]. Although SBV was the major component of the viral inoculum, IAPV replicated to higher levels than other viruses [29]. However, it turned out that the AmE-711 cell line utilized was no longer available to repeat the experiments, and moreover it was persistently infected with DWV [29,30]. *A. ceranae* primary cells were infected with SBV and sustained SBV replication [31]. However, an important disadvantage of using primary cells resides in the fact that their development is time-consuming; after a relatively short period of time, the cells die and are no longer available, which requires fresh primary cultures that need to be validated for their biological behavior [30,31].

Given the substantial difficulties encountered to develop an *A. mellifera* reliable cell-culture system to study honey bee virus infections, we wanted to encourage the exploration of the ability of available cell lines from heterologous insects (many of them reviewed in [32]) to sustain honey bee virus replication, as was demonstrated for the Lepidopteran cell line IPL-LD-65Y and the microbial pathogens of *A. mellifera*, *Nosema apis*, and *ceranae* [33]. For this purpose, we used a Lepidopteran hemocytic cell line (P1) and evaluated its ability to support infection with DWV. We show that DWV was able to infect P1 cells. Moreover, a tagged-primer-based RT-PCR analysis showed the presence of the negative-sense DWV RNA in the cells, indicating the replication of the virus. However, it appeared that DWV from infected cells was poorly infectious to P1 cells as well as to *A. mellifera* pupae.

## 2. Materials and Methods

### 2.1. Cells and Viruses

**Figure 1:**
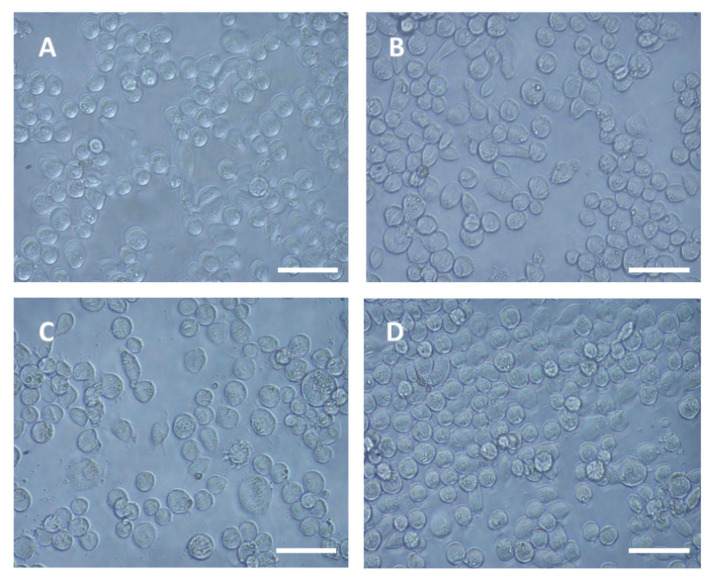
Light micrograph of Deformed wing virus (DWV)-infected and mock-infected P1 cells. DWV-infected cells at 0, 24, and 48h post-infection (panels **A**, **B**, and **C**, respectively); **D**, mock-infected cells at 48 h. Bar 50 µm.

The P1 Lepidopteran cell line was subcloned from the SPC-PL 65 hemocytic cell line from *Spodoptera litura* [34]; adapted to grow in TNM-FH insect medium (Sigma-Aldrich, Israel) supplemented with fetal bovine serum (10%), Penicillin, and Streptomycin antibiotics; and passaged for over 25 years (Figure 1d). For virus infections, the cells were seeded in a 12-well plate at a density of 8 × 10^5^/well [35] and incubated overnight at 28 °C.

DWV: the virus inoculum consisted of a highly DWV-enriched fraction prepared from *Varroa destructor*-infected mites (described in [36]). Varroa mites (600 from 7 honey bee colonies) were ground to homogeneity in sodium phosphate (0.01 M, pH 7, containing 0.02% sodium diethyldithiocarbamate). The sample was clarified from tissue debris by 10 min centrifugation at 800× *g*, 4 °C, in a Hettich Universal 32R centrifuge (Hettich Lab Technology, Tuttlingen, Germany), followed by a second clarification at 10,000× *g* for 5 min, 4 °C in a microcentrifuge (Select BioProducts, Edison, NJ, USA). The clarified supernatant was overlaid to a solution of 30% sucrose in phosphate-buffered saline pH 7.5 (PBS) and subjected to 4 h (h) of ultracentrifugation at 100,000× *g*, 4 °C, in a Sorvall Discovery 90SE ultracentrifuge (Thermo Fisher Scientific, Waltham, MA. USA) using a TY35 rotor (Hitachi, Tokyo, Japan). The viral pellet was resuspended in 500 µL of PBS and utilized for infections. An RT-qPCR analysis revealed that the DWV inoculum was composed of more than 99.99% DWV-A and -B, IAPV (Israeli acute paralysis virus) 0.0001%, VDV-2 (Varroa destructor virus-2) 0.0004%, and VDV-3 (Varroa destructor virus-3) 0.0001% [36].

The infection of P1 cells was performed by carefully removing the cell media, followed by addition of 300 µL of infectious medium containing DWV in TNM-FH medium without serum (DWV 2.53 × 10^8^ genomic copies). Since the cell doubling time is about 40 h, the estimated multiplicity of infection was 200 DWV genomic copies per cell. At 30 min post-infection (p.i), the viral inoculum was removed, the cells were briefly washed in virus-free medium, and new fresh medium with 5% FBS was added. The cells were further incubated at 28 °C until they were harvested for RNA extraction at the times indicated in the relevant experiments. Before the RNA extraction, the supernatants containing the putative DWV-P1 virus were collected and frozen at −80 °C to serve as inocula for subsequent infections performed in the study.

### 2.2. Cell Harvesting and RNA Extraction

At various times post infection (p.i.), the cells and their supernatant were harvested, transferred to microcentrifuge tubes (Eppendorf AG, Hamburg, Germany), and centrifuged at 2500× *g* rpm for 5 min, 4 °C. The cell supernatants containing putative infectious DWV (DWV-P1) were transferred to new Eppendorf tubes and kept at −80 °C for infectivity tests. The RNA extraction of the cells was performed with BioTri ™ (Bio-Lab Ltd., Jerusalem, Israel) following the manufacturer’s instructions.

### 2.3. RT-qPCR

cDNA was prepared using RevertAid Reverse Transcriptase (Thermo Fisher Scientific) with oligo-dT and random primers according to the manufacturer’s instructions. One thousand nanograms of RNA templates were used. The RT-conditions were the incubation of RNA and primers at 65 °C for 5 min, followed by the addition of buffer containing 50 mM of Tris-HCl (pH 8.3), 75 mM of KCl, 2 mM of MgCl2, 5 mM of DTT, 4 units of RNase inhibitor Ribolock^®^ (Thermo Fisher Scientific), and the RT enzyme (200 units) in a 25 µL volume, and further incubation at 55 °C for 30 min. The reaction was terminated by heating at 85 °C for 5 min.

The viral genome copy number was quantified on a PikoReal 96 machine (Thermo Fisher Scientific) using a standard protocol (95 °C for 2 min; 40 cycles of 95 °C for 10 s, 60 °C for 20 s, and 72 °C for 20 s). Each quantitative PCR analysis was performed in triplicate. Non-template controls (water) were included in triplicate for each assay. The KAPA SYBR^R^ FAST qPCR Master Mix (2×) Universal (Kapa Bio-systems, Wilmington, MA, USA) was used in a 10 µL final volume. For each analysis, 2 µL of the diluted cDNA (250 ng/µL) were used (dilution factor = 4). The DWV primers used were 6138F and 6326R (at 0.25 µM), and the housekeeping primers used were RPL8 F and RPL8 R [37]. The DWV primers used are described in Supplementary Table S1.

For P1 cell infections, the increase in the number of DWV copies relative to the start of the infection after washing the viral inoculum was calculated as follows:

ΔCt = ΔCt target (DWV)-ΔCt reference gene (housekeeping); ΔΔCt = ΔCt − ΔCt calibrator (Ct DWV at 0.5 h after the removal of inoculum and washing the cells); the DWV relative quantities were calculated as 2^−ΔΔCt^.

It should be noted that the values obtained for the expression of the housekeeping gene did not change throughout the DWV infection of the P1 cells.

The genomic equivalents of DWV were calculated as follows:

Two calibration curves were prepared for DWV and the housekeeping gene.

Amplicons of 209 bp containing the DWV target sequence were obtained by performing PCR with the primers 6138F and 6326R (Supplemental Materials Table S1). A 10-point standard curve was prepared (four-fold serial dilutions of the obtained amplicon with known concentrations from 4 pg (Cq = 8.5) and up to 1.5 × 10^−5^ pg (Cq = 27.5)). The specificity of the amplicons synthesized during the PCR run was ascertained by performing a dissociation curve protocol from 60 to 95 °C. The efficiency of the PCR reaction was E = 99%, R^2^ = 0.9979, and the slope = −3.349. The housekeeping primers RPL8 F and RPL8 R were used to set up a calibration curve (E = 99.787%; y = −0.301x + 3.306; R^2^ = 0.9986), as described before [37,38] (primers in Supplementary Table S1). The DWV loads of 100 ng total RNA extracted from the individual samples were calculated by plotting the Ct values against the logarithm of the RNA copy number using the PikoRealTM Software 2.2 (Thermo-Fisher Scientific, Waltham, MA, United States). These values were used to calculate the DWV genomic copy number in the RNA extracted from the individual sample after normalization to the housekeeping gene.

Statistical analyses was performed with a one-way analysis of variance (ANOVA) using JMP Pro 14.

### 2.4. DWV Replication Test

The testing for DWV viral replication (presence of the negative strand-sense RNA) was performed as described before [39] by synthesizing the complementary cDNA with the tagged primer tag-6138F. Subsequently the residual tag-6138F primer was inactivated by adding it to the mixture exonuclease-I (Exo-I) and incubating it for another 15 min at 37 °C [7]. Finally, Exo-I was inactivated by heating the mixture at 80 °C for 15 min. The PCR was performed subsequently with the primers DWV 6326R and tag (Supplementary Table S1). To ensure that any remaining tag-6138F primers were left after the cDNA reaction, the PCR was performed using only the 6326R primer. Additionally, the cDNA produced without any primer was used as a control in the same reactions, followed by PCR with the same primers as above. The PCR was performed at 94 °C for 4 min, with 30 cycles at 94 °C for 30 s, then at 58 °C for 50 s, at 72 °C for 30 s, and a final extension step of 72 °C for 10 min. The identity of the amplified fragment (225 nucleotides) was confirmed by conventional Sanger sequencing (at the Biological Services Unit of the Weizmann Institute of Science, Israel).

### 2.5. Inoculation of A. mellifera Pupa with DWV-P1

*A. mellifera* red-eyed pupa were taken out from a brood comb of a Varroa-treated colony. The pupa were inoculated using a Hamilton microliter syringe (USA) with 1 µL of DWV-P1 (infected cell supernatant collected at various times p.i.), mock-infected cell supernatant, or heat-inactivated DWV supernatant collected from cells diluted in sterile phosphate buffer saline (PBS) at pH 7.5. The inoculation was performed by inserting the syringe needle between the second and third abdominal segments of the pupae. The uninjected pupae served as an additional control throughout the experiment. The pupae were placed in sterile petri-dishes and in an incubator at 32 °C with a 35% humidity. In the first experiment, a total of 30 pupae were tested and divided into six groups of 5 pupa each. One group was not injected at all. The other five groups were individually injected as follows: two groups were injected with the supernatant collected from cells at 0.5 h and 48 h, respectively. Another group was injected with heat-inactivated DWV, and the last group was injected with mock-infected cell supernatant. In the second experiment, a larger number of pupae was used (64 pupae, *n* = 16 per treatment, 4 groups), plus an additional group of 4 pupae that were injected with the original DWV inoculum (positive control). Two groups were injected with the supernatant collected from cells at 0.5 h and 48 h, respectively. Another group was injected with mock-infected cell supernatant, and the last one was not injected at all. The pupae were collected at 72 h post-injection for RNA extraction and RT-qPCR analysis. The RNA from each pupa was extracted with BioTri ™ (Bio-Lab Ltd., Jerusalem, Israel), following the manufacturer’s instructions as above, and the absolute amount of DWV genomic copies was calculated, as described above.

Statistical analysis was performed with a one-way analysis of variance (ANOVA) using JMP Pro 14.

## 3. Results and Discussion

### 3.1. DWV Replicates in P1 Cells

To study the ability of DWV to infect P1 cells, the cells were seeded in 12-well plates and, after overnight incubation at 28 °C, were overlaid with the virus inoculum, as described in Materials and Methods. The swelling of the cells was observed at 24 h post infection and it increased by 48 h; fewer cells were observed in comparison with the mock-infected cells plated as controls at 48 h (Figure 1, compare panels A, B, C, and D). However, we could not observe an extensive cytopathic effect and cell lysis.

**Figure 2:**
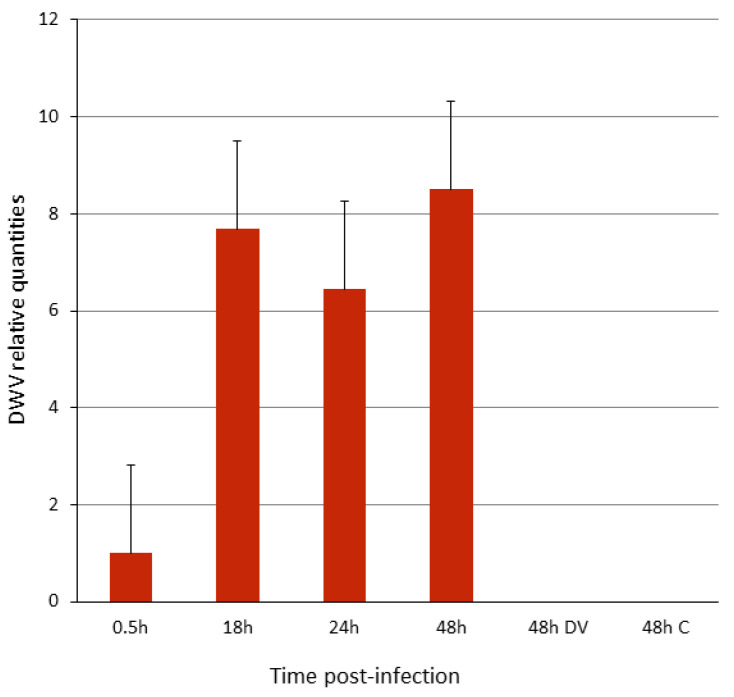
Infection of P1 cells with DWV. *Y*-axis, relative amount of DWV genomic copies, normalized expression in respect to the virus bound to the cells for 0.5 h of incubation and wash. *X*-axis, h, hours; DV, heat-inactivated virus inoculum; C, mock-infected cells (see Materials and Methods, Section 2.3). Bars represent standard error values. One-way ANOVA (F = 4.8581, P = 0.041).

To investigate whether DWV entered and infected the cells, they were harvested at various times post-infection, the RNA was extracted, and the number of DWV genomic copies per sample was determined by a RT-quantitative PCR using DWV-specific primers. The amount of DWV genomic copies increased to about 8-fold at 18 h p.i. and continued to increase at 48 h p.i. relative to the virus bound/associated with the cells at 0.5 h p.i. (Figure 2). At 48 h post-treatment, the cells infected with heat-inactivated DWV-inoculum (DV, pre-heated at 85 °C for 10 min) or mock-infected (C) did not show DWV genomic copies (Figure 2, ibid.).

**Figure 3:**
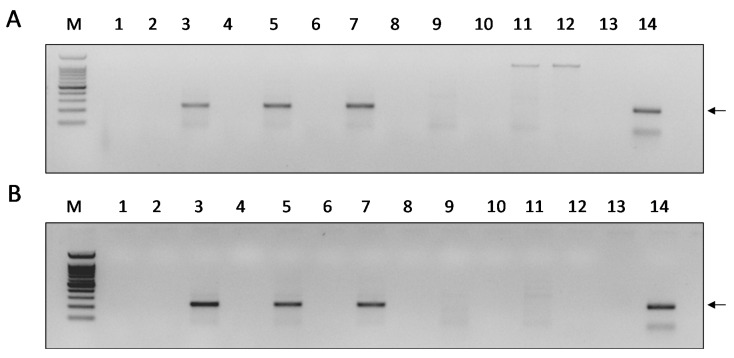
Detection of the DWV negative-sense RNA strand in infected P1-cells. Panels **A** and **B**, PCR from cells incubated for 15 and 30 min with DWV and subsequent wash of the viral inoculum, respectively. Lanes 1 and 2—Samples extracted at 15 and 30 min p.i. (Panels A and B, respectively). Lanes 3–4, 5–6, 7–8—Samples extracted at 18, 24, and 48 h. p.i., respectively. Lanes 9–10—samples extracted from heat-inactivated DWV-infection at 48 h p.i. Lanes 11–12—Extracts from mock-infected cells. Even numbers indicate a PCR control reaction from the same individual RNA performed on the cDNA produced without any primer (see the section Materials and Methods, Section 2.4). M—100 bp DNA Ladder RTU Marker (GeneDireX^TM^), lower band 100 bp. Lane 13—NTC, non-template control, amplification of the positive sample without PCR primers. Lane 14, DWV amplicon (arrow).

To validate that the increase measured in the DWV-genomic copy number was the result of the production of new DWV genomes, we tested for DWV replication in the P1-infected cells by monitoring the presence of its negative-sense RNA-strand. For this purpose, we used RNA-strand sense-specific primer-tagged-RT-PCR (see DWV replication test in Materials and Methods). A predicted fragment of 225 nucleotides corresponding to the DWV negative-sense RNA-strand size (comprised between nucleotides 6138 and 6347 of the viral genome with the addition of the tag) was found at 18, 24, and 48 h p.i. (Figure 3, panels A and B, lanes 3, 5, and 7, respectively). Thus, this indicated that the increase in DWV genomic copies observed was due to the virus replication. No amplification was observed when the cells were extracted and tested at 15 or 30 min p.i. (Figure 3, panels A and B lanes 3, 5, and 7), or at 48 h p.i. using heat-inactivated DWV inoculum (Figure 3, panels A and B, lane 9), or in the mock-infected control samples (Figure 3, panels A and B, lane 11). Similar results were obtained when the cDNAs were prepared from the RNA of these samples without an oligonucleotide primer in the RT reaction, and the PCR was performed subsequently, as described above (Figure 3, A and B, even-numbered lanes). To ensure that no rests of the tag-6138F primers were left after the cDNA reaction, the same PCR was performed using only the 6326R primer (please see Supplementary Material, Figure S1).

### 3.2. Infectivity of DWV-P1

**Figure 4:**
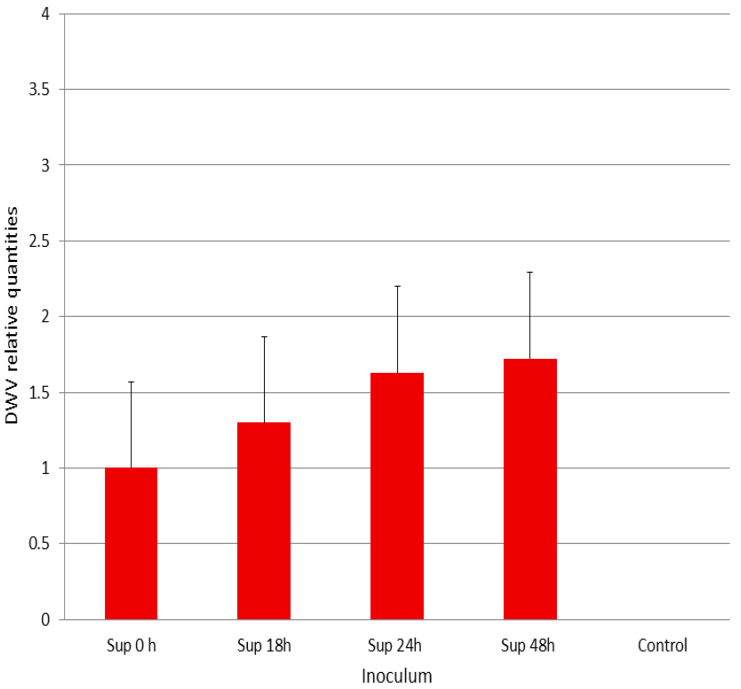
Infectivity of DWV-P1 to P1 cells. *Y*-axis, relative amount of DWV genomic copies, normalized expression in respect to the virus bound to the cells for 0.5 h of incubation and wash (Sup. 0). *X*-axis, supernatant from P1 DWV-infected cells (DWV-P1) collected at various hours (h) p.i. used as inoculum. Control, supernatant from mock-infected cells used as inoculum. Bars represent standard error values. One-way ANOVA (F = 0.6954, P = 0.6333).

Since DWV replicated in P1 and despite the fact that the infected cells did not show extensive lysis, we decide to test the infectivity of their supernatant (DWV-P1) to fresh P1 cells and honey bees. For this purpose, freshly seeded P1 cells were infected with the supernatants collected from the experiment described above (see Section 3.1 and Materials and Methods, Section 2.2). At 24 h p.i., the cells were harvested and the relative amount of DWV genomic copies was determined, as described in the previous section. As can be seen in Figure 4, the DWV-P1 inoculum from 18, 24, and 48 h supernatants collected in the previous round of infection generated relatively more DWV genomic equivalents than those collected at 0.5 h that were used as the reference (Figure 4). However, this increase was not statistically significant (one-way ANOVA F = 0.6954, P = 0.6333).

To examine the possibility that the DWV generated in the first round of infection of P1 cells (DWV-P1) was infectious to *A. mellifera*, we injected the supernatant collected at 48 h p.i. from the experiments in Section 3.1 to honey bee pupae that were selected from DWV-negative colonies (see Materials and Methods, Section 2.5 and Supplementary Material Tables S2 and S3, column “uninfected”). As controls, we used 48 h p.i. (DWV-P1) supernatant from heat-inactivated DWV-infected cells, the supernatant of mock-infected P1 cells, as well as uninfected pupae. This was performed in two separate sets of experiments.

**Figure 5:**
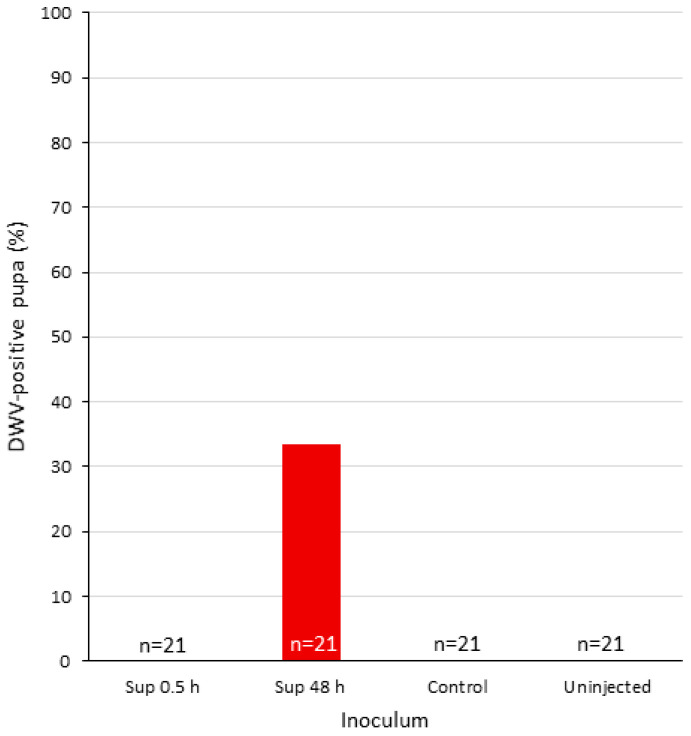
DWV prevalence of injected pupae. *X*-axis, individual pupae treated as follows: Injected with DWV-P1 at 0.5 and 48 h (Sup. 0.5 and 48 h, respectively) supernatant from mock-infected cells (control) or uninjected (see Materials and Methods, Section 2.5).

Seven out of 21 pupa injected with the DWV-P1 inoculum from 48 h were positive for DWV (33.3% prevalence, Figure 5), with a range of 6.8 × 10^2^ to 1.46 × 10^10^ genomic copies, a significant difference from the 47 injected pupa of the control groups described above that were negative (see Supplementary Material, Figure S2 and Tables S2 and S3). All of the 21 uninjected pupa were negative as well (Figure 5 and Supplemental Material, Figure S2 and Tables S2 and S3, ibid.). Four pupae inoculated with the original DWV inoculum were positive, with genomic equivalents between 8.41 × 10^6^ to 1.43 × 10^10^ (Supplementary Material, Table S3).

The low level of DWV-P1 infectivity to P1 cells and prevalence in *A. mellifera* pupae (33.33% of the injected bees) may be due the fact that few infectious DWV particles were released from the infected P1 cells, since we could not detect an extensive cytopathic effect and cell lysis (Figure 1). It is possible that, after an initial burst of infection, the cells were able to manage and convert it to a persistent infection. In this context, it is worth noting that Carrillo-Trip et al. reported that DWV persistently infected the AmE-711 *A. mellifera* cells [29]. DWV is known for its ability to persist in *A. mellifera* in a silent asymptomatic state [4]. Moreover, RNA viruses were reported to establish persistent infections in Lepidopteran cells [40,41].

It is also conceivable that very few viral capsids were produced or that the viral polyprotein was poorly processed. However, the fact that we observed the replication of DWV in P1 suggests that the viral polyprotein was processed to yield functional viral peptides. It has been proposed that RNA-mediated interference and reverse transcription control the persistence of RNA viruses in Drosophila [42]. If this is the case in P1 cells infected with DWV, the introduction of a viral suppressor of RNAi (via transfection or via co-infection with a suppressor-bearing virus) could lead to the production of a higher number of DWV infectious particles, as has been shown by Carrillo-Trip and coworkers [29].

This study shows that DWV can replicate in a heterologous cell line. This is a first step, since the virus yield from the infected cells appeared to be very low. Thus, more research is required to improve the virus yields in this cell line. Given the availability of many other insect cell lines [30,32], our study paves the way for future exploration in this direction. In the absence of adequate *A. mellifera* cell lines, exploring the ability of alternative cell lines to enable honey bee virus infections could provide the means to study and understand the viral infectious cycle at the cellular level and facilitate obtaining purified isolates of these viruses.

## Supplementary Materials

The following are available online at https://www.mdpi.com/1999-4915/12/7/739/s1: Figure S1: Detection of the DWV negative-sense RNA-strand in infected P1-cells by sense-specific primer-tagged-RT-PCR, exo-1 control. Figure S2: DWV genomic copies in injected pupa with statistical analysis. Mean DWV genomic copies of the treatments of DWV-injected pupa. Table S1: Primers used in qPCR and to monitor DWV replication. Tables S2 and S3: Infectivity of DWV-P1 to *A. mellifera*. Exps. 1 and 2, respectively.

## References

[1] Beaurepaire A., Doublet V., De Miranda J.R., Piot N., Antunez K., Campbell E., Chantawannakul P., Chejanovsky N., Gajda A., Heerman M. (2020). Diversity and global distribution of viruses of the western honey bee, Apis mellifera. Insects.

[2] Chen Y.P., Siede R. (2007). Honey bee viruses. Adv. Virus Res..

[3] De Miranda J.R., Cordoni G., Budge G. (2009). The Acute bee paralysis virus—Kashmir bee virus—Israeli acute paralysis virus complex. J. Invertebr. Pathol..

[4] De Miranda J.R., Genersch E. (2010). Deformed wing virus. J. Invertebr. Pathol..

[5] De Miranda J.R., Dainat B., Locke B., Cordoni G., Berthoud H., Gauthier L., Neumann P., Budge G.E., Ball B.V., Stoltz D.B. (2010). Genetic characterization of slow bee paralysis virus of the honeybee (*Apis mellifera* L.). J. Gen. Virol..

[6] Ribière M., Olivier V., Blanchard P. (2010). Chronic bee paralysis: A disease and a virus like no other?. J. Invertebr. Pathol..

[7] De Miranda J.R., Bailey L., Ball B.V., Blanchard P., Budge G.E., Chejanovsky N., Chen Y., Gauthier L., Genersch E., De Graaf D.C. (2013). Standard methods for virus research in Apis mellifera. J. Apic. Res..

[8] Chen Y.P., Pettis J.S., Corona M., Chen W.P., Li C.J., Spivak M., Visscher P.K., DeGrandi-Hoffman G., Boncristiani H., Zhao Y. (2014). Israeli acute paralysis virus: Epidemiology, pathogenesis and implications for honey bee health. PLoS Pathog..

[9] Hartmann U., Forsgren E., Charrière J.D., Neumann P., Gauthier L. (2015). Dynamics of Apis mellifera filamentous virus (AmFV) infections in honey bees and relationships with other parasites. Viruses.

[10] Genersch E., Von der Ohe W., Kaatz H., Schroeder A., Otten C., Büchler R., Berg S., Ritter W., Mühlen W., Gisder S. (2010). The German bee monitoring project: A long term study to understand periodically high winter losses of honey bee colonies. Apidologie.

[11] Runckel C., Flenniken M.L., Engel J.C., Ruby J.G., Ganem D., Andino R., DeRisi J.L. (2011). Temporal analysis of the honey bee microbiome reveals four novel viruses and seasonal prevalence of known viruses, Nosema, and Crithidia. PLoS ONE.

[12] Soroker V., Hetzroni A., Yakobson B., David D., David A., Voet H., Slabezki Y., Efrat H., Levski S., Kamer Y. (2011). Evaluation of colony losses in Israel in relation to the incidence of pathogens and pests. Apidologie.

[13] Cornman R.S., Tarpy D.R., Chen Y., Jeffreys L., Lopez D., Pettis J.S., VanEngelsdorp D., Evans J.D. (2012). Pathogen webs in collapsing honey bee colonies. PLoS ONE.

[14] Hou C., Rivkin H., Slabezki Y., Chejanovsky N. (2014). Dynamics of the presence of israeli acute paralysis virus in honey bee colonies with colony collapse disorder. Viruses.

[15] Cavigli I., Daughenbaugh K.F., Martin M., Lerch M., Banner K., Garcia E., Brutscher L.M., Flenniken M.L. (2016). Pathogen prevalence and abundance in honey bee colonies involved in almond pollination. Apidologie.

[16] Amiri E., Meixner M., Büchler R., Kryger P. (2014). Chronic bee paralysis virus in honeybee queens: Evaluating susceptibility and infection routes. Viruses.

[17] Thaduri S., Stephan J.G., De Miranda J.R., Locke B. (2019). Disentangling host-parasite-pathogen interactions in a varroa-resistant honeybee population reveals virus tolerance as an independent, naturally adapted survival mechanism. Sci. Rep..

[18] Hunter W., Ellis J., Vanengelsdorp D., Hayes J., Westervelt D., Glick E., Williams M., Sela I., Maori E., Pettis J. (2010). Large-scale field application of RNAi technology reducing Israeli acute paralysis virus disease in honey bees (Apis mellifera, Hymenoptera: Apidae). PLoS Pathog..

[19] Lamp B., Url A., Seitz K., Eichhorn J., Riedel C., Sinn L.J., Indik S., Köglberger H., Rümenapf T. (2016). Construction and rescue of a molecular clone of Deformed wing virus (DWV). PLoS ONE.

[20] Ryabov E.V., Christmon K., Heerman M.C., Posada-Florez F., Harrison R.L., Chen Y., Evans J.D. (2020). Development of a honey bee RNA virus vector based on the genome of a deformed wing virus. Viruses.

[21] Jin L., Mehmood S., Zhang G., Song Y., Su S., Huang S., Huang H., Zhang Y., Geng H., Huang W.F. (2020). Visualizing sacbrood virus of honey bees via transformation and coupling with enhanced green fluorescent protein. Viruses.

[22] Gusachenko O.N., Woodford L., Balbirnie-Cumming K., Campbell E.M., Christie C.R., Bowman A.S., Evans D.J. (2020). Green bees: Reverse genetic analysis of deformed wing virus transmission, replication, and tropism. Viruses.

[23] Oldstone M.B.A., Levine A.J. (2000). Virology in the Next Millennium. Cell.

[24] Carrillo-Tripp J., Bonning B.C., Miller W.A. (2015). Challenges associated with research on RNA viruses of insects. Curr. Opin. Insect Sci..

[25] Adelman Z.N., Sanchez-Vargas I., Travanty E.A., Carlson J.O., Beaty B.J., Blair C.D., Olson K.E. (2002). RNA silencing of dengue virus type 2 replication in transformed C6/36 mosquito cells transcribing an inverted-repeat RNA derived from the virus genome. J. Virol..

[26] Swevers L., Liu J., Smagghe G. (2018). Defense mechanisms against viral infection in Drosophila: RNAi and non-RNAi. Viruses.

[27] Franco E.J., Rodriquez J.L., Pomeroy J.J., Hanrahan K.C., Brown A.N. (2018). The effectiveness of antiviral agents with broad-spectrum activity against chikungunya virus varies between host cell lines. Antivir. Chem. Chemother..

[28] Goblirsch M.J., Spivak M.S., Kurtti T.J. (2013). A cell line resource derived from honey bee (Apis mellifera) embryonic tissues. PLoS ONE.

[29] Carrillo-Tripp J., Dolezal A.G., Goblirsch M.J., Miller W.A., Toth A.L., Bonning B.C. (2016). In vivo and in vitro infection dynamics of honey bee viruses. Sci. Rep..

[30] Guo Y., Goodman C.L., Stanley D.W., Bonning B.C. (2020). Cell lines for honey bee virus research. Viruses.

[31] Xia X., Mao Q., Wang H., Zhou B., Wei T. (2014). Replication of Chinese sacbrood virus in primary cell cultures of Asian honeybee (Apis cerana). Arch. Virol..

[32] Genersch E., Gisder S., Hedtke K., Hunter W.B., Möckel N., Müller U. (2013). Standard methods for cell cultures in Apis mellifera research. J. Apic. Res..

[33] Gisder S., Mockel N., Linde A., Genersch E. (2011). A cell culture model for Nosema ceranae and Nosema apis allows new insights into the life cycle of these important honey bee-pathogenic microsporidia. Environ. Microbiol..

[34] Li Y., Jousset F.-X., Giraud C., Rolling F., Quiot J.M., Bergoin M. (1996). A titration procedure of the Junonia Cœnia Densovirus and quantitation of transfection by Its cloned genomic DNA in four lepidopteran cell lines. J. Virol. Methods.

[35] Chejanovsky N., Gershburg E. (1995). The wild-type Autographa californica nuclear polyhedrosis virus induces apoptosis of Spodoptera littoralis cells. Virology.

[36] Levin S., Sela N., Chejanovsky N. (2016). Two novel viruses associated with the Apis mellifera pathogenic mite Varroa destructor. Sci. Rep..

[37] Zioni N., Soroker V., Chejanovsky N. (2011). Replication of varroa destructor virus 1 (VDV-1) and a varroa destructor virus 1-deformed wing virus recombinant (VDV-1-DWV) in the head of the honey bee. Virology.

[38] Evans J.D. (2006). Beepath: An ordered quantitative-PCR array for exploring honey bee immunity and disease. J. Invertebr. Pathol..

[39] Levin S., Galbraith D.A., Sela N., Erez T., Grozinger C.M., Chejanovsky N. (2017). Presence of apis rhabdovirus-1 in populations of pollinators and their parasites from two continents. Front. Microbiol..

[40] Ma H., Nandakumar S., Bae E.H., Chin P.J., Khan A.S. (2019). The Spodoptera frugiperda Sf9 cell line is a heterogeneous population of rhabdovirus-infected and virus-negative cells: Isolation and characterization of cell clones containing rhabdovirus X-gene variants and virus-negative cell clones. Virology.

[41] Merten O.W. (2002). Virus contaminations of cell cultures—A biotechnological view. Cytotechnology.

[42] Goic B., Vodovar N., Mondotte J.A., Monot C., Frangeul L., Blanc H., Gausson V., Vera-Otarola J., Cristofari G., Saleh M.C. (2013). RNA-mediated interference and reverse transcription control the persistence of RNA viruses in the insect model Drosophila. Nat. Immunol..

