# Supplementary material for: Infection of a Lepidopteran Cell Line with Deformed Wing Virus
Source: Viruses. 2020 Jul 9;12(7):739. doi: 10.3390/v12070739 (PMC7412015; doi:10.3390/v12070739)
Supplement: Supplementary file 1 [file viruses-12-00739-s001.pdf]

Supplementary Materials

Figure S1: Detection of the DWV negative-sense RNA strand in infected P1-cells by sense-specific primer-tagged-RT-PCR, exo-1 control

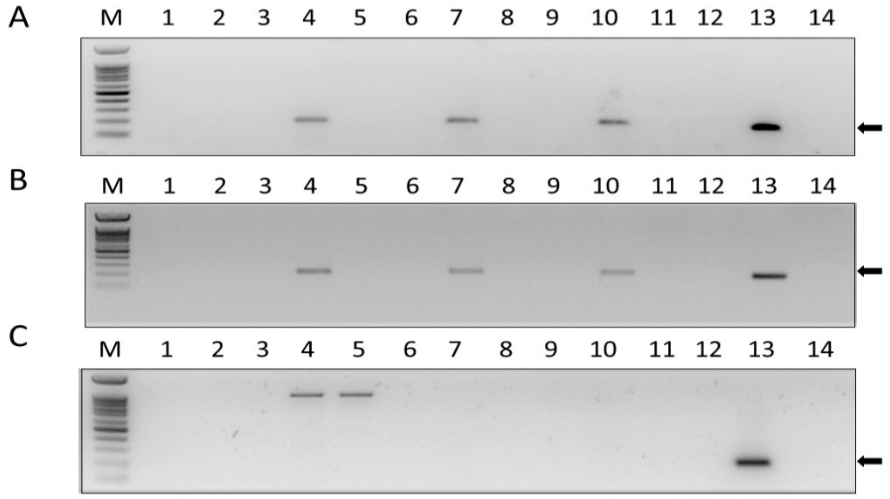

Detection of the DWV negative-sense RNA strand in infected P1-cells by sense-specific primer-tagged-RT-PCR, exo-1 control. Panels A and B, PCR from cells incubated for 15 and 30 min with DWV and subsequent wash of the viral inoculum, respectively: lanes 1, 2 and 3 samples extracted at 15 and 30 min p.i., respectively; lanes 4, 5, and 6, samples extracted at 18 h p.i.; lanes 7, 8 and 9 samples extracted at 24 h p.i.; lanes 10, 11, and 12 samples extracted at 48 h. p.i., respectively. Lanes 2, 5, 8, and 11 indicate PCR control reaction from the same individual RNA performed on cDNA produced without any primer; lanes 3, 6, 9 and 12 indicate PCR reaction from the same individual RNA performed with the reverse primer 6326R only (see the section "Materials and Methods). M, 100 bp DNA Ladder RTU Marker (GeneDireX™). Lane 13, DWV, positive control amplicon (also indicated by the arrow). Lane 14, NTC, amplification of positive sample without PCR primer. Panel C. Cells infected with heat-inactivated DWV infection at 48 h p.i and mock-infected cells. Lanes 1, 2, 3 and 7, 8, 9, samples incubated for 15 and 30 min with heat-inactivated DWV and subsequent wash of the viral inoculum, respectively; lanes 4, 5, 6 and 10, 11, 12, samples extracts from mock-infected cells. Lanes 2, 5, 8, and 11 PCR control reaction from the same individual RNA performed on cDNA produced without any primer; lanes 3, 6, 9 and 12 indicate PCR reaction from the same individual RNA performed with the reverse primer 6326R only (see the section "Materials and Methods). M, 100 bp DNA Ladder RTU Marker (GeneDireX™). Lane 13, DWV, positive control amplicon (also indicated by the arrow). Lane 14, NTC, amplification of positive sample without PCR primer.

Table S1: primers utilized in qPCR and to monitor DWV replication

Primer	Sequence
DWV: 6138F	TGGCTAACCGTCGTAAGGCG
DWV: 6326R	TAACTGACGCACTAATTTCCGC
tag-6138F	AGCCTGCGCACCGTGGTGGCTAACCGTCGTAAGGCG
tag	AGCCTGCGCACCGTGG
RPL8 F (honey bee, housekeeping)	TGGATGTTCAACAGGGTTCATA
RPL8 R (honey bee, housekeeping)	CTGGTGGTGGACGTATTGATAA

1000 ng of RNA extracted from P1 cells infected with DWV was used in qRT-PCR reactions with the various primers described above.

Table S2. Infectivity of DWV-P1 to *A. mellifera. Exp.1*

Injected Pupae (#)	DWV-P1 source: Sup. p.i. 0.5 h	DWV-P1 source: Sup. p.i. 48 h	DWV-P1 source: Heat-inactivated inoculum	Sup. Control	Uninfected
	DWV genomic copies detected				
1	0	0	0	0	0
2	0	0	0	0	0
3	0	2.1×10^3	0	0	0
4	0	6.8×10^2	0	0	0
5	0	0	0	0	0

arbitrary number of individual pupa that were injected with the above described inoculum (each column corresponds to one kind of treatment per serial numbered pupae): DWV-P1 source-, Heat-inactivated DWV, Control, or Uninfected (untreated). Genomic copies of DWV were measured by RT-qPCR using the primers 6138F+6326R.

Table S3. Infectivity of DWV-P1 to *A. mellifera. Exp.2*

Injected Pupae (#)	DWV-P1 source: Sup. p.i. 0.5 h	DWV-P1 source: Sup. p.i. 48 h	Sup. Control	Uninfected	DWV Positive control*
	DWV genomic copies detected				
1	0	0	0	0	1.43×10^{10}
2	0	0	0	0	3.03×10^{9}
3	0	0	0	0	7.18×10^{7}
4	0	1.62×10^{9}	0	0	8.41×10^{6}
5	0	0	0	0	
6	0	1.46×10^{10}	0	0	
7	0	0	0	0	
8	0	1.98×10^{5}	0	0	
9	0	4.44×10^{5}	0	0	
10	0	0	0	0	
11	0	0	0	0	
12	0	5.14×10^{7}	0	0	
13	0	0	0	0	
14	0	0	0	0	
15	0	0	0	0	
16	0	0	0	0	

arbitrary number of individual pupa that were injected with the above described inoculum (each column corresponds to one kind of treatment per serial numbered pupae): DWV-P1 source-, Control, or Uninfected (untreated). *DWV positive control, injection of original DWV inoculum used to infect the cells. Genomic copies of DWV were measured by RT-qPCR using the primers 6138F+6326R.

Figure S2: DWV genomic copies in injected pupa with statistical analysis. Mean DWV genomic copies of the treatments of DWV-injected pupa. Data from Tables S4 and S5.

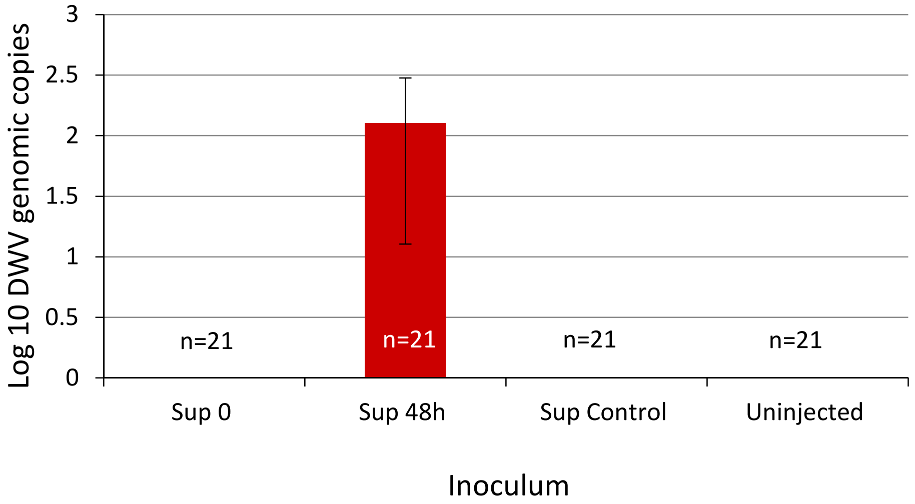

Data from Tables S2 and S3. Bars represent standard error values. One-way ANOVA ($F = 7.9585$, $P = 0.0001$).
